# Next Generation Exon 51 Skipping Antisense Oligonucleotides for Duchenne Muscular Dystrophy

**DOI:** 10.1089/nat.2022.0063

**Published:** 2023-06-02

**Authors:** Judith van Deutekom, Chantal Beekman, Suzanne Bijl, Sieto Bosgra, Rani van den Eijnde, Dennis Franken, Bas Groenendaal, Bouchra Harquouli, Anneke Janson, Paul Koevoets, Melissa Mulder, Daan Muilwijk, Galyna Peterburgska, Bianca Querido, Janwillem Testerink, Ruurd Verheul, Peter de Visser, Rudie Weij, Annemieke Aartsma-Rus, Jukka Puoliväli, Timo Bragge, Charles O'Neill, Nicole A. Datson

**Affiliations:** ^1^ VICO Therapeutics B.V., formerly BioMarin Nederland B.V., Leiden, The Netherlands.; ^2^ Department of Human Genetics, Leiden University Medical Centre, Leiden, The Netherlands.; ^3^ Charles River Discovery Services, Kuopio, Finland.; ^4^ BioMarin Pharmaceutical, Inc., San Rafael, California, USA.

**Keywords:** antisense oligonucleotide, exon skipping, dystrophin, Duchenne muscular dystrophy

## Abstract

In the last two decades, antisense oligonucleotides (AONs) that induce corrective exon skipping have matured as promising therapies aimed at tackling the dystrophin deficiency that underlies the severe and progressive muscle fiber degeneration in Duchenne muscular dystrophy (DMD) patients. Pioneering first generation exon 51 skipping AONs like drisapersen and eteplirsen have more recently been followed up by AONs for exons 53 and 45, with, to date, a total of four exon skipping AON drugs having reached (conditional) regulatory US Food and Drug Administration (FDA) approval for DMD. Nonetheless, considering the limited efficacy of these drugs, there is room for improvement. The aim of this study was to develop more efficient [2′-*O*-methyl-modified phosphorothioate (2′OMePS) RNA] AONs for *DMD* exon 51 skipping by implementing precision chemistry as well as identifying a more potent target binding site. More than a hundred AONs were screened in muscle cell cultures, followed by a selective comparison in the hDMD and hDMDdel52/*mdx* mouse models. Incorporation of 5-methylcytosine and position-specific locked nucleic acids in AONs targeting the drisapersen/eteplirsen binding site resulted in 15-fold higher exon 51 skipping levels compared to drisapersen in hDMDdel52/*mdx* mice. However, with similarly modified AONs targeting an alternative site in exon 51, 65-fold higher skipping levels were obtained, restoring dystrophin up to 30% of healthy control. Targeting both sites in exon 51 with a single AON further increased exon skipping (100-fold over drisapersen) and dystrophin (up to 40%) levels. These dystrophin levels allowed for normalization of creatine kinase (CK) and lactate dehydrogenase (LDH) levels, and improved motor function in hDMDdel52/*mdx* mice. As no major safety observation was obtained, the improved therapeutic index of these next generation AONs is encouraging for further (pre)clinical development.

## Introduction

Antisense oligonucleotides (AONs) designed to specifically induce exon skipping during pre-messenger RNA (pre-mRNA) splicing have shown to be effective in increasing expression of (truncated) dystrophin in a variety of cell and mouse models for Duchenne muscular dystrophy (DMD) [1–9]. DMD is a chromosome X-linked, severe, and progressive neuromuscular disease caused by the lack of sufficient levels of functional dystrophin, primarily in muscle and brain [10,11]. Despite the large number of studies and publications in the field, to date, it still is not clearly defined precisely how much dystrophin restoration is necessary to yield a functional improvement in DMD patients with a more or less advanced disease status [12].

Levels of (lifelong expression of) dystrophin in Becker muscular dystrophy (BMD) patients have been reported to vary between 10 and 90%, with a mean of 33% [13,14], but these may not be easily obtainable when starting therapies targeting dystrophic muscle tissue later in life. There are currently four AONs (conditionally) approved by the US Food and Drug Administration (FDA): (1) eteplirsen (Exondys51) inducing exon 51 skipping (<1% dystrophin of normal reported in muscle tissue after 188 weeks of treatment) [15–18], (2) golodirsen (Vyondys53) inducing exon 53 skipping (<1% dystrophin of normal reported after 48 weeks of treatment) [19], (3) casimersen (Amondys45) inducing exon 45 skipping (up to 1.7% dystrophin of normal reported after 48 weeks of treatment) [20,21], and (4) viltolarsen (Viltepso), also inducing exon 53 skipping (<5.9% dystrophin of normal reported after 25 weeks of treatment, although measured with a different methodology compared to the aforementioned studies) [22].

The small increases in dystrophin expression obtained with these AONs justify further efforts to develop next generation oligonucleotides with improved pharmacokinetic and pharmacodynamic properties. Increased oligonucleotide circulation time, biodistribution, cellular uptake and/or target duplex stability can be obtained by adjusting target sequence, length, chemical composition, and/or conjugation to cell-penetrating or muscle-homing peptides or antibodies. A lot of information on aforementioned parameters has been collected from the Prosensa Therapeutics- and Sarepta Therapeutics-sponsored (pre)clinical studies between 2006 and 2016 on the first generation exon 51 skipping AONs drisapersen and eteplirsen (Exondys51) [16,17,23–26].

**FIG. 1.**
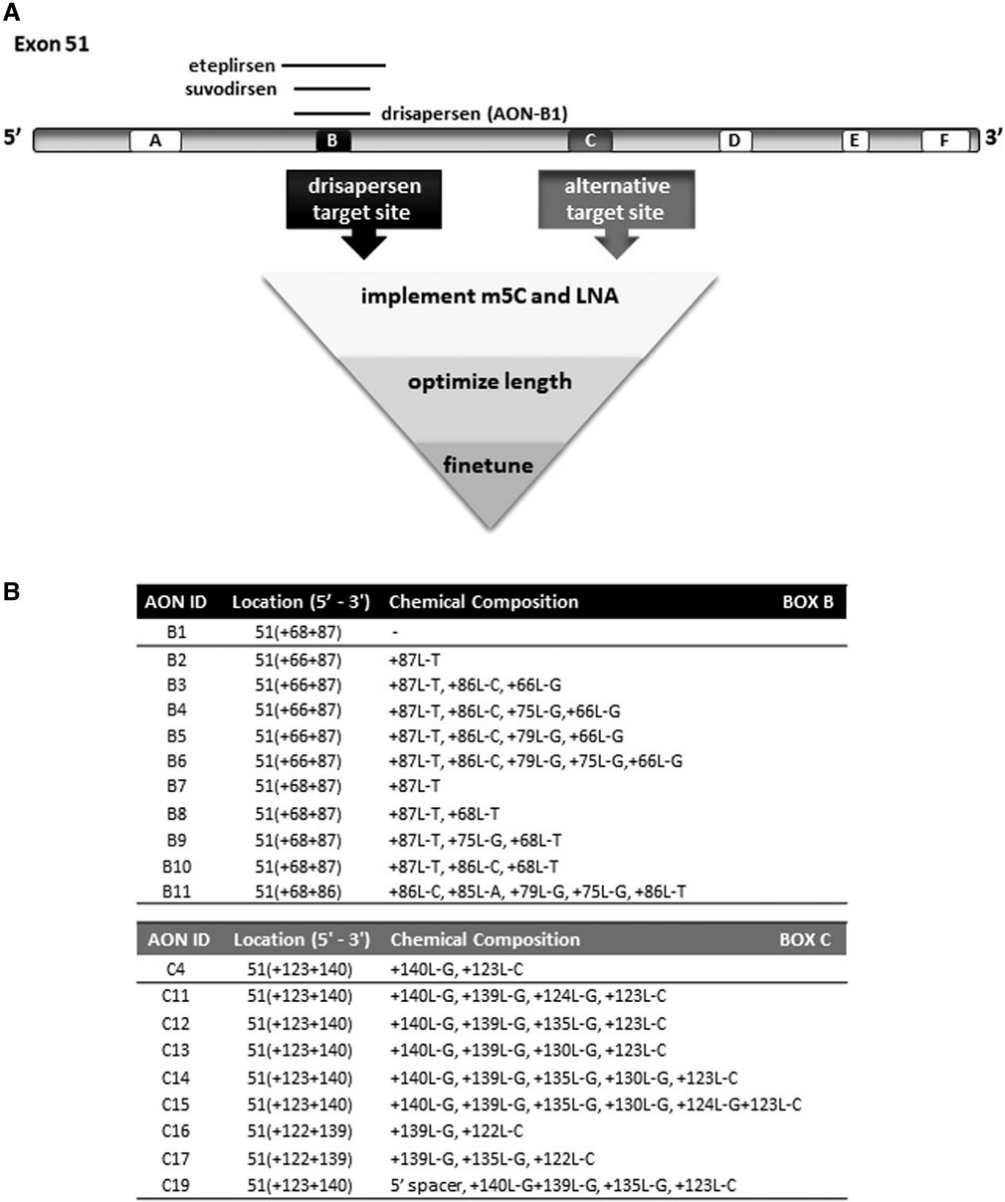
Overview of target sites and AONs for *DMD* exon 51. **(A)** Exon 51 with the location of boxes A–F representing putative splicing regulatory elements as predicted by the RESCUE-ESE Web Server and/or ESEfinder 3.0 software packages. Box B is the shared target for AONs known in the field: eteplirsen (Exondys 51), drisapersen (AON-B1), and suvodirsen. Box C is here identified as a novel, alternative and highly potent target site for AONs inducing higher levels of exon 51 skipping. The other boxes A, D, E, and F appeared not to be involved in splicing and are thus not effective target sites. **(B)** Specific target location of AONs that are highlighted in this article, with LNA base and position details per AON. All AONs, except AON-B1, contained 5-methylcytosines. AONs, antisense oligonucleotides; DMD, Duchenne muscular dystrophy; L = LNA, locked nucleic acid; L-A, adenine-LNA; L-C, cytosine-LNA; L-G, guanine-LNA; L-T, thymine-LNA.

Although differing in length and chemical composition [2′-*O*-methyl-modified phosphorothioate (PS) RNA and phosphorodiamidate morpholino oligomer, respectively], both compounds target the same sequence stretch (+68 + 87) in exon 51 (Fig. 1), which was already identified by Aartsma-Rus *et al*. in 2002 [27]. A third AON, suvodirsen [a 2′-fluoro-2′-deoxy-modified phosphorothioate (Sp)/phosphodiester RNA] [28], recently withdrawn from further clinical development by Wave Life Sciences, also targeted this same sequence.

The aim of this study was to apply lessons learned from over a decade of (pre)clinical *DMD* exon skipping studies, by including a more extensive screening of potential target sites in exon 51, a larger panel of precision chemistry 2′-*O*-methyl-modified phosphorothioate (2′OMePS) RNA AONs, a more thorough, funnel-like, AON candidate screening, not only in muscle cell cultures but especially also in clinically relevant humanized DMD mouse models, and a selection of most efficient AONs on the basis of a sufficient sample/cohort size and sensitive and quantitative RNA and protein assays.

Our starting point was drisapersen, a 20-mer 2′OMePS RNA oligonucleotide. Although multiple other next-generation oligonucleotide backbone and sugar chemistries have been considered by us and others, like morpholino phosphorodiamidate (PMO), peptide nucleic acid (PNA), tricycloDNA (tcDNA), stereodefined phosphorothioate (PS), methylphosphonate, and 2′-Fluoro (2′F), or 2′-*O*-methoxyethyl (2′MOE) [29], none of these has, to date, shown to be a convincing improvement over 2′OMePS.

Two chemical modifications that are compatible with 2′OMePS and have consistently shown interesting potential are 5-methylpyrimidines and bridged nucleic acids. More specifically, implementation of naturally existing 5-methylcytosines is known to increase AON-RNA target duplex stability and/or reduce immune stimulation [29–32]. Adding specifically positioned locked nucleic acid (LNA) nucleotides further increases not only AON-RNA duplex stability but also enzymatic stability (especially at the 3′ and 5′ termini) [29,31,32]. Both modifications are off-patent, commercially available, and easy to implement.

As the 19 PS linkages in drisapersen may have contributed to some of the safety observations in clinical studies, we also reduced AON length down to 16 nucleotides. We thus screened a large series of drisapersen-derivative AONs (Fig. 1) and selected those with an at least 10-fold higher efficiency, which would allow for lower clinical dosing regimen based on an increased benefit-risk profile. To search for potentially even more efficient AONs, we also re-screened the entire exon 51 sequence for alternative target stretches that may be more dominantly involved in exon 51 splicing (Fig. 1). The effectiveness of double targeting, by combining two oligonucleotides hybridizing to separate target sequences, was also explored.

In total, more than a hundred 2′OMePS oligonucleotides were designed and first tested for *in vitro* efficacy in DMD patient myotube cultures. We applied the state-of-the-art droplet digital polymerase chain reaction (ddPCR) and capillary Western immunoassay technologies to identify AON candidates inducing the highest levels of exon 51 skipping and dystrophin expression *in vitro*. These were then further evaluated *in vivo* in the hDMD and hDMDdel52/*mdx* mouse models [8,33–35]. The latter model contains mutated murine and human *DMD* genes, and therefore lacks both mouse and human dystrophin. It has a motor deficit and allows for preclinical screening, at both the molecular and functional level, of human-specific AON drug candidates inducing the skipping of human *DMD* exon 51. Three lead AONs with a strongly improved therapeutic index relative to drisapersen were identified as interesting candidates for further (pre)clinical development.

## Materials and Methods

### Antisense oligonucleotides

All AONs described herein consisted of 2′-*O*-methyl PS RNA and were synthesized by BioMarin Nederland B.V. (Leiden, The Netherlands).

### Cell culture experiments

Immortalized DMD patient-derived myoblasts [Δ48–50; kindly provided by the Association Institut de Myology (AIM)] [36]) were cultured to confluency in six-well plates. To induce the formation of myotubes, proliferation medium [skeletal muscle cell growth medium (No. C23060, including supplementary pack; PromoCell), 20% heat-inactivated fetal bovine serum (FBS) (No. 10270-106; Invitrogen), and Penicillin-Streptomycin 10,000 U/mL (No. 15140-122; Invitrogen)], was replaced on day 0 by low-serum differentiation medium [Dulbecco's modified Eagle's medium, DMEM (No. 11880-028; Invitrogen), 2% heat-inactivated FBS (No. 10270-106; Invitrogen), Glucose 45% (No. G8769; Sigma-Aldrich), GlutaMAX Supplement (No. 35050-038; Invitrogen), and Penicillin-Streptomycin 10,000 U/mL (No. 15140-122; Invitrogen)] for 7 or 21 days.

**FIG. 2.**
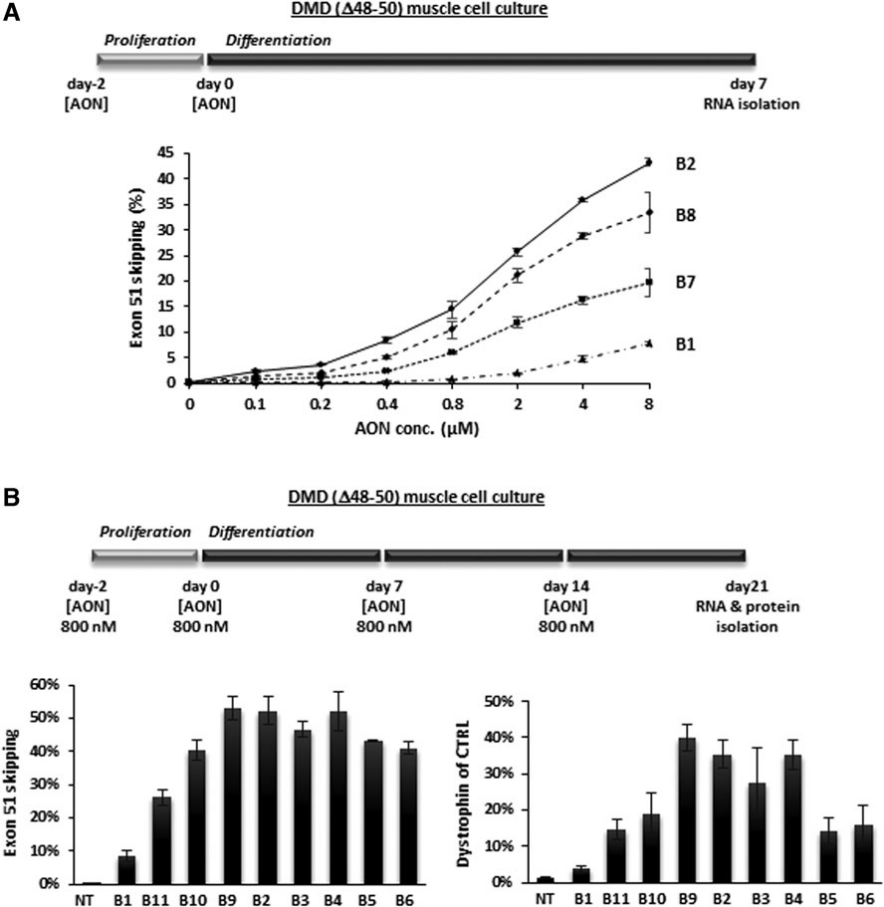
*In vitro* screening of AONs targeting box B in exon 51 through gymnotic uptake in muscle cell cultures from a DMD patient with a deletion of exons 48–50. **(A)** Experimental setup for our standard screening of AONs: two administration time points on days −2 and 0, followed by RNA isolation on day 7. At increasing AON concentrations from 0.1 to 8 μM, exon 51 skipping levels were assessed by RT-ddPCR: skip % of total *DMD* transcripts, mean of *n* = 3 ± SD. **(B)** Experimental setup of a longer term screening: four administration time points on days −2, 0, 7, and 14, followed by RNA and protein isolation on day 21. *Left graph*: exon 51 skipping levels (RT-ddPCR, skip % of total *DMD* transcripts, mean of *n* = 6 ± SD); *right graph*: dystrophin levels (WES, % of healthy control muscle cell culture, mean of *n* = 6 ± SD). RT-ddPCR, reverse transcriptase-droplet digital polymerase chain reaction; NT, nontreated cells; SD, standard deviation; WES, Simple Western Analysis.

AON was added to the medium to allow gymnotic uptake: on days −2 and 0 at increasing concentrations of 0.1–8 μM (see schedule Fig. 2A), or on days −2, 0, 7, and 14 at a fixed concentration of 800 nM (see schedule Fig. 2B). Total RNA was extracted from the cultures using 500 μL/well NucleoZol (No. 740404.200; Macherey Nagel), according to the manufacturer's instructions. Complementary DNA (cDNA) was generated in 20 μL reactions, using 1,000 ng of total RNA with 3.2 μg random hexamer primers (No. 11034731001; Roche) and GoScript (No. A5003; Promega), according to the manufacturer's instructions.

Protein lysates were prepared by adding 75 μL Protein Lysis Buffer [15% SDS, 75 mM Tris-HCl pH 6.8, 20% glycerol, and 1 Protease Inhibitor Cocktail tablet (No. 04693159001; Roche/Sigma)/8 mL, 6.25% β-Mercaptoethanol] per well, scraping the bottom of the wells and collecting the content in 1.5 mL vials. Cells were disrupted by passing the lysate through a 21 G needle 15 times, and then they were spun down briefly (20 s) and stored at −80°C until further use. To measure total protein concentration, 20 × dilutions of lysates in H_2_O were measured using the Pierce 660 nm Protein assay (No. 226607; Thermo Scientific) with added Ionic Detergent Compatible Reagent (22663; Thermo Scientific), according to the manufacturer's instructions.

### Mouse husbandry and ethical approval

All animal experiments were performed at Charles River Laboratories (CR) (Finland). The hDMD [33,34], hDMDdel52/*mdx* [8,35], and C57BL6/J mouse colonies were housed in IntraCage Ventilation cages at a density of four-five mice per cage in a temperature- and humidity-controlled environment with a normal light/dark cycle. Food (Purina Lab Diet 5001) and water were available *ad libitum*. Experiments were executed as specified in the license authorized by the national Animal Experiment Board of Finland and according to the National Institutes of Health (Bethesda, MD) guidelines for the care and use of laboratory animals.

### Treatment of mice with different AONs

For all hDMD and hDMDdel52/*mdx* mouse studies, mice were randomized into groups, taking body weight and gender distribution into account. The correct genotype of the hDMDdel52/*mdx* mice was confirmed before enrolment. Five- to seven-week-old mice were treated by 12 or 13 weekly intravenous (IV) injections in the tail vein. Doses varied between 9 and 100 mg/kg equimolar to AON-B1. Control groups of 10–20 wild-type (C57BL6/J), hDMDdel52/*mdx* or hDMD mice of the same age as the AON-treated mice were included for the serum biomarkers and motor function testing and received weekly IV injections with vehicle (VEH 20 mM sodium phosphate buffer pH 7 with 0.7% w/v NaCl).

Before each dose, mice were weighed. Mice were sacrificed 4 (hDMD) or 14 (hDMDdel52/*mdx*) days after the final injection, by deep anesthetization with sodium pentobarbital (60 mg/kg Mebunat; Orion Pharma), followed by cardiac puncture. A blood sample was collected to measure serum biomarkers, after which the mice were transcardially perfused with phosphate-buffered saline (PBS). Quadriceps muscle, heart, kidney, and liver were isolated and snap-frozen by immersing in isopentane au-bain-marie in liquid nitrogen, placed in cryovials prechilled on dry ice, and stored at −80°C.

### ddPCR analysis of exon skip levels

Human dystrophin exon 51 skipping was determined using ddPCR [37]. Total RNA was isolated from quadriceps muscle or heart tissues homogenized in 1 mL Nucleozol (No. 740404.200; Macherey Nagel) by homogenizing in a MagNa Lyser (2–4 cycles of 20 s at 7,000 rpm and cooling on ice after 2 cycles) using MagNA Lyser Green Beads (No. 03358941001; Roche). The RNA concentration was determined with Nanodrop. cDNA was generated with 1,000 ng total RNA in 20 μL reactions using 3.2 μg random hexamer primers (No. 11034731001; Roche) and GoScript (No. A5003; Promega) according to the manufacturer's instructions, with the exception that random hexamer primer incubation took place for 10 min at 65°C instead of 5 min at 70°C and reverse transcription was performed at 50°C for 40 min.

Eighty units of enzyme per reaction were used in a final concentration of 8 mM MgCl and 1 mM dNTPs each. Specific TaqMan assays were designed to detect the dystrophin exon 50–52 or 50–53 boundary (transcripts with exon 51 skipped in hDMD or hDMDdel52/*mdx*, respectively) and the exon 50–51 boundary (transcripts still containing exon 51, referred to as nonskipped). ddPCR was performed as previously described [9,37].

### Simple Western Analysis of dystrophin restoration

Protein lysates were prepared from snap-frozen skeletal and heart muscle biopsies, and analyzed on a Simple Western Analysis (WES) system (No. 004-600; ProteinSimple) as previously described [9,13]. For dystrophin detection, a rabbit monoclonal anti-dystrophin antibody (No. ab154168, dilution 1/1,000; Abcam) was applied. An antibody targeting vinculin (No. Vinculin E1E9V, 13901S, dilution 1/100; Cell Signalling) was included to control for sample loading. As both primary antibodies were raised in rabbit, an anti-rabbit secondary antibody (No. 042-206; ProteinSimple) was used. For vinculin, this anti-rabbit-HRP antibody was diluted with 1/1,000 unconjugated anti-rabbit antibody (No. ab6702; Abcam) to reduce HRP activity.

Six-point calibration curves of a previously characterized wild-type C57BL6/10ScSn mouse muscle lysate were included, ranging from 0.008 to 0.25 μg for dystrophin, and from 0.25 to 4.0 μg for vinculin. The final vinculin-corrected dystrophin values were generated by first expressing both the dystrophin and vinculin signals as percentage of control [% wild-type (WT) calculated from the calibration curve] and then dividing the dystrophin % WT value by the vinculin % WT value.

### Hybridization-ligation assay for measuring AON concentration

The concentration of AONs was determined using a full-length product-specific sandwich hybridization enzyme-linked immunosorbent assay (ELISA) method. Tissues were homogenized as previously described [9]. Tissue samples (60 mg/mL) were diluted 1/60 in PBS and further dilutions were made in blank tissue matrix [1/60 diluted pooled control tissue (60 mg/mL) in PBS]. AON-specific biotinylated capture probes (25 nM phosphodiester DNA oligonucleotides with LNA modifications (in capital letters): gaacttacC-biotin for AON-C12, atcttcctTGA-biotin for AON-C18 and -C19 [Eurogentec]) were added to streptavidin-coated 96-well plates (Roche), incubated at 37°C for 30 min, and then washed four times with TBST. Samples were added and incubated at 37°C for 30 min, followed by a wash step (4 × ) with TBST.

Digoxigenin (DIG)-conjugated AON-specific detection probes (2 nM phosphodiester DNA oligonucleotides with LNA modifications (in capital letters): digoxigenin-GCttggaca for AON-C12, digoxigenin-gcTTGgacaga for AON-C18, and digoxigenin-gcTTGgacaga for AON-C19 [Eurogentec]) were added and incubated at 45°C for 30 min, followed by a wash step (4 × ) with TBST. Anti-DIG POD (Roche) in blocking buffer (1% milk in TBST) was added and incubated for 30 min in the dark at room temperature, followed by a wash step (4 × ) with TBST. Following addition of 3,3′,5,5′-tetramethylbenzidine (TMB; Sigma) and incubation for a maximum of 30 min, the reaction was stopped by the addition of maleic acid (345 mM; Sigma). Absorption was directly measured at 450 nm using a plate reader. Calibration curves and QC samples of the AON prepared in blank tissue matrix were included. All analyses were performed in duplicate.

### Histopathology

In the hDMDdel52/*mdx* mouse study focusing on AON-12, AON-18, and AON-19, samples of liver, kidney, spleen, lymph nodes, heart, and skeletal muscle were collected (from at least five males and five females per group) at necropsy and fixed in 7% neutral buffered formalin. The formalin-fixed samples were processed to hematoxylin and eosin-stained slides for microscopic evaluation by Charles River Laboratories France Safety Assessment. The incidence of microscopic findings was scored and severity grades (from minimal, mild, moderate to severe) were assigned.

### Hematology

In the hDMDdel52/*mdx* mouse study focusing on AON-12, AON-18, and AON-19, EDTA whole blood samples were collected for routine hematology, including hemoglobin, red blood cell count, white blood cell count, absolute reticulocyte count, reticulocyte %, and thrombocyte count.

### Clinical chemistry

In the hDMDdel52/*mdx* mouse study focusing on AON-12, AON-18, and AON-19, unhemolyzed serum samples were collected for routine clinical chemistry, including alanine aminotransferase (ALT), aspartate aminotransferase (AST), alkaline phosphatase (ALP), gamma-glutamyl transferase (GGT), creatine kinase (CK), and lactate dehydrogenase (LDH).

### Fine motor and kinematic gait analysis

In the hDMDdel52/*mdx* mouse study focusing on AON-12, AON-18, and AON-19, fine motor skills and gait properties were assessed at baseline (week 6), mid-dosing after 8 doses (week 14), and 1 week after the 13th and final dose (week 20) in *n* = 12–20 mice per group using a high-precision kinematic analysis method (MotoRater; TSE Systems, Homburg, Germany, https://criver.widen.net/s/glcsfpnz5l) using the walking mode, as previously described [9,38]. Different gait patterns and movements were further analyzed using a custom-made automated analysis system. The analyzed parameters included general spatiotemporal parameters, body posture, balance, and fine motor skills.

The analysis provided altogether 95 different parameters related to fine motor capabilities and gait. The correlation structure between these distinctive parameters was further assessed using principal component analysis (PCA). A set of new uncorrelated parameters, the principal components, was determined. Finally, an Overall Gait Score, based on PCA of kinematic data, was established. The basis of this score, the identified discriminant vector, is a disease model-specific combination of original variables, which characterizes the model in the best possible way. The Overall Gait Score for each mouse was obtained by projecting the normalized parameter data onto the discriminant vector.

### Statistical analysis

All values were presented as mean ± standard deviation, and differences were considered to be statistically significant at the *P* < 0.05 level. Significance between groups was assessed by analysis of variance and Tukey's multiple comparisons test (*P < 0.05; **P < 0.01;***P < 0.001; ****P < 0.0001).

## Results

### Design of AONs

All 2′OMePS RNA AONs in this study were subjected to a multi-phase funnel screening, from DMD patient cell cultures to mouse models, to filter out the most efficient candidates for further preclinical evaluation. A summary and the highlights of this large assessment are described in this article. We followed two lines of investigation, not only focusing on drisapersen but also reconsidering alternative exon 51 target sites (Fig. 1).

Using drisapersen (henceforth referred to as AON-B1), a 20-mer 2′-*O*-methyl PS RNA, and its target sequence in exon 51 (box B in Fig. 1) as starting point, we first evaluated the effect of implementing chemical modifications, including 5-methylcytosines (throughout the AON) and LNA nucleotides (5′- and/or 3′-terminal positions only). To further increase the AON-RNA duplex stability, longer versions of AON-B1 (with or without one or more LNAs) were designed, and shorter versions with more (internal) LNAs (but less than 5 to ensure sequence specificity and safety).

Next, a precision chemistry approach was applied, in which specific base variants and positions of the LNAs (terminal and internal) (Fig. 1B) were further compared and optimized. Although the aimed threshold of at least 10-fold improvement over drisapersen (AON-B1) was reached, we also re-screened exon 51 for potentially more potent splice-regulatory elements as alternative target sites (boxes A, C–F in Fig. 1). These were identified by the RESCUE-ESE Web Server [39] and/or ESEfinder 3.0 [40,41] software packages. Per site, a series of overlapping AONs was tested initially. Only one other target (box C), just downstream of the one for drisapersen (box B), was found effective and a similar AON design optimization path was taken. The most efficient AONs were selected and subjected to further fine-tuning of the specific position of the LNA(s) (Fig. 1B).

### *In vitro* screening of AONs targeting box B in DMD patient (del 48–50) myotube cultures

The primary *in vitro* AON selection threshold was set at ≥10-fold higher exon skip levels when compared to AON-B1 in immortalized DMD patient (del 48–50) muscle cell cultures. A large series of standard gymnotic uptake experiments was conducted. In the first screening focusing on AON-B1 variants between 16 and 22 nucleotides, all with m5C and one or two 5′ and/or 3′ terminal LNAs, the majority of AONs was found 2- to 10-fold more effective than AON-B1. The improved efficiency of shorter variants (16- to 19-mers) in particular confirmed the hypothesized positive effect of LNAs on increasing AON-RNA duplex stability. The longer AONs (20- to 22-mers) were, however, even more effective. As an example, Fig. 2A shows a concentration series experiment (from 0.1 to 8 μM) applying an experimental setup, wherein AON-B2, a 22-mer with one 5′ thymine-LNA, was most efficient over the entire range of tested concentrations.

At the lower concentrations AON-B2 resulted in an improvement of up to 23-fold over AON-B1, and at the highest concentration tested of 8 μM, it induced 43.3% exon 51 skipping compared to 7.8% for AON-B1. The shorter AON-B7 and AON-B8 variants of AON-B2, sharing the same 20 nucleotide sequence as AON-B1, also exhibited markedly increased skipping efficiencies compared to AON-B1 (at 8 μM 19.7% and 33.4%, respectively), although less than AON-B2. The additional 3′ thymine-LNA in AON-B8 clearly contributed to its more positive efficiency relative to AON-B7.

The second series of AON-B1 variants, of which a selection is shown in Fig. 2B, focused on 19- to 22-mers, 5′- and/or 3′-terminal LNAs, plus additional internal LNAs (guanine- and cytosine-LNAs in particular). These AONs, including AON-B2 from Fig. 2A, were compared to AON-B1 in a longer term gymnotic uptake experiment in which the AONs were administered to the cells at a lower concentration (800 nM) four times on days −2, 0, 7, and 14. AON-B1 induced 8.7% exon 51 skipping, resulting in 3.8% dystrophin of normal (from a healthy muscle cell culture). AON-B9 was most efficient with 53.1% exon skipping and 40.0% dystrophin of normal.

This 20-mer AON, isosequential to AON-B1, has a 5′ and 3′ terminal thymine-LNA and an additional guanine-LNA at a specific position in the central part. AON-B4, a 22-mer, was second best with two 5′ terminal LNA's, one 3′ terminal LNA, and the same guanine-LNA position as AON-B9. There was no additional effect of its increased length, or its second 5′ terminal cytosine-LNA compared to AON-B9, suggesting a dominant effect of that single internal guanine-LNA position. Although this guanine-LNA was also present in AON-B11 and AON-B6, both AONs were less efficient than AON-B4 and AON-B9, possibly due to a countereffect of a second internal guanine-LNA just upstream. Remarkably, AON-B2, although with just one 5′-terminal thymine-LNA, was in the top 4 of most efficient AONs. In general, the levels of exon 51 skipping correlated well with the dystrophin levels (*R*^2^ = 0.9321).

### *In vivo* screening of AONs targeting box B in the hDMD and hDMDdel52/*mdx* mouse models

The ten most efficient box B AON candidates selected from the *in vitro* comparative screening assays (shown in Fig. 2) were further evaluated and compared to AON-B1 in the hDMD transgenic mouse model [33,34]. This mouse model contains the full-length human *DMD* gene integrated in a mouse autosome, and expresses both mouse and human dystrophin. Therefore, the hDMD model is not a disease model of dystrophin deficiency, but nonetheless can be used to study whether human-specific AONs can induce exon 51 skipping at the mRNA level, although at low levels due to the limited uptake of AONs by healthy (nondystrophic) muscle fibers. In two subsequent studies, cohorts of 15 hDMD mice (M/F) received 12 weekly IV (tail vein) injections of 100 mg/kg AON-B1 or an equimolar amount of the selected AON candidates.

**FIG. 3.**
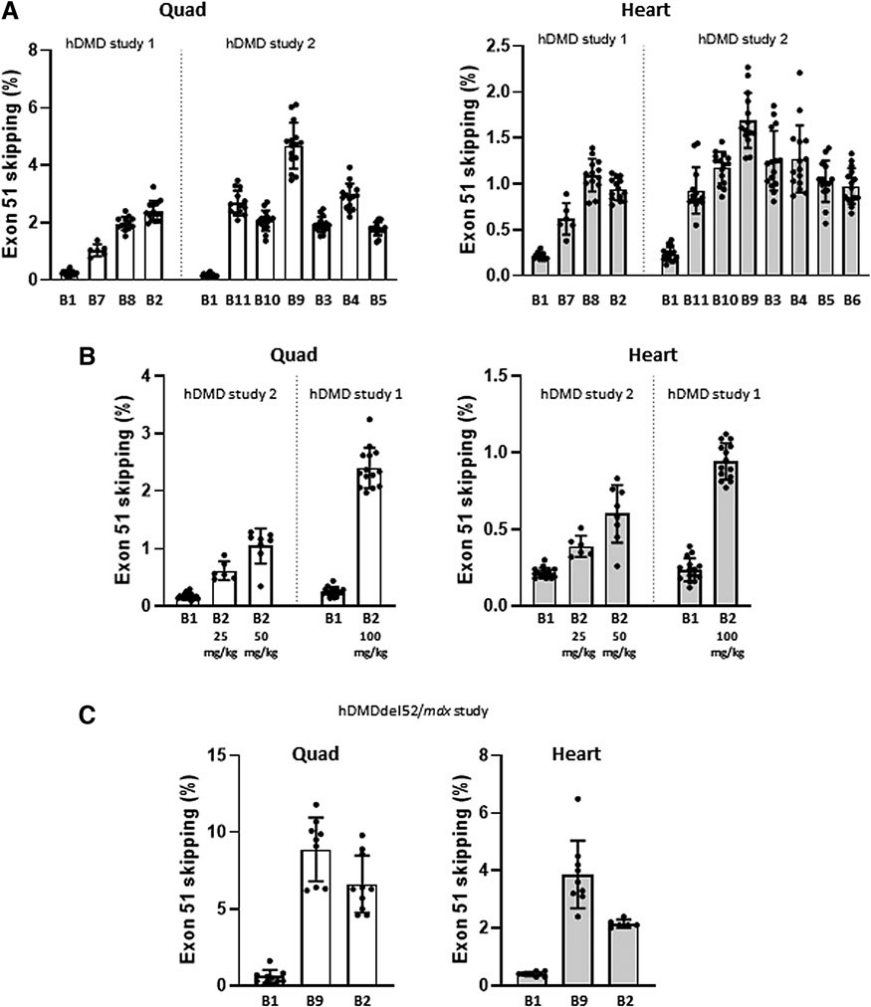
*In vivo* screening of AONs targeting box B in hDMD and hDMDdel52/*mdx* mouse models. **(A, B)** Exon 51 skipping levels in quadriceps and heart of hDMD mice. **(C)** Exon 51 skipping levels in quadriceps and heart of hDMDdel52/*mdx* mice. RT-ddPCR, skip % of total *DMD* transcripts (mean ± SD). *Dots* represent values obtained in individual mice, after receiving full treatment (12/12 doses). Due to tail necrosis, not all mice reached the endpoint. Note: the *Y*-axis scale of the graphs is different for quadriceps and heart to focus on the differences between the AONs in these tissues. Overall, the exon 51 skipping levels in heart were approximately three times lower than in quadriceps.

This dose regimen has previously been used to administer the M23D AON, and was effective in inducing exon 23 skipping of mouse dystrophin in *mdx* mice, while also being well tolerated [9]. At 4 days after the last injection, RNA was isolated from quadriceps and heart and analyzed by reverse transcriptase-droplet digital polymerase chain reaction (RT-ddPCR). In the first study AON-B7, AON-B8, and AON-B2 (from Fig. 2A) were compared to AON-B1. AON-B2 was most efficient (2.4% exon 51 skipping in quadriceps), 10-fold more than AON-B1 (0.25%) (Fig. 3A, left graph). In the heart, highest exon 51 skipping levels were obtained with AON-B8 (1.1% compared to 0.22% with AON-B1).

In a second larger study, the AON candidates from Fig. 2B were included. AON-B9 was most efficient with 4.7% exon 51 skipping in quadriceps, which is 27-fold better than AON-B1 (Fig. 3A, right graph). In the heart, AON-B9 induced 1.7% exon 51 skipping compared to 0.24% with AON-B1 (7-fold improvement). The other AON candidates in this second hDMD study were all at least as efficient as AON-B2 (up to 3% exon 51 skipping). For AON-B2, three increasing doses were tested in these studies; equimolar to AON-B1: 25 or 50 mg/kg in study 2 and 100 mg/kg in study 1. A clear dose–response was observed in both quadriceps and the heart (Fig. 3B).

AON-B2 (the 22-mer with the lowest number of LNAs (1)) and AON-B9 (the most efficient 20-mer) were then selected for further evaluation in the hDMDdel52/*mdx* mouse model [8,35]. This mouse model carries the human dystrophin gene with an exon 52 deletion in an *mdx* background, resulting in a dystrophin deficiency and associated motor deficits. It thus not only allows preclinical evaluation of human-specific AONs inducing the skipping of exon 51 in dystrophic muscle tissue but also assessment of restoration of dystrophin synthesis and resulting effect on motor performance. Cohorts of 10 hDMDdel52/*mdx* mice (M/F) received 12 weekly IV (tail vein) injections. RNA was isolated from quadriceps and heart 10 days after the last injection. AON-B2 was again administered at a dose equimolar to 100 mg/kg AON-B1. For the twofold more efficient AON-B9, a lower dose was explored (equimolar to 60 mg/kg AON-B1), and compared to AON-B1 at that same dose.

The levels of exon 51 skipping in mice treated with AON-B2 were up to 6.7% in quadriceps and 2.2% in the heart (Fig. 3C). These levels are over twofold higher than those obtained at the same dose in hDMD mice (Fig. 3A), which confirms the better uptake of AONs by dystrophic muscle tissue. Despite the lower dose, AON-B9-treated hDMDdel52/*mdx* mice showed even higher exon skipping efficiencies: 8.9% in quadriceps and 3.9% in the heart, 15-fold over the obtained AON-B1 efficiencies (0.6% and 0.4%, respectively). Although these results were encouraging and met a minimal threshold of 10-fold improvement in efficacy over AON-B1, we nevertheless decided to search for potentially even more efficient AONs by targeting an alternative target sequence in exon 51.

### *In vitro* screening of AONs targeting box C in DMD patient (del 48–50) myotube cultures

An AON walk was performed across exon 51, focusing on alternative regions encompassing putative splicing regulatory motifs identified by RESCUE-ESE Web Server and ESEfinder 3.0 [39–41] (boxes A, C to F in Fig. 1A). Per box, a series of overlapping AONs were screened at 800 nM in DMD patient (del 48–50) myotube cultures (similar experimental setup as in Fig. 2A).

**FIG. 4.**
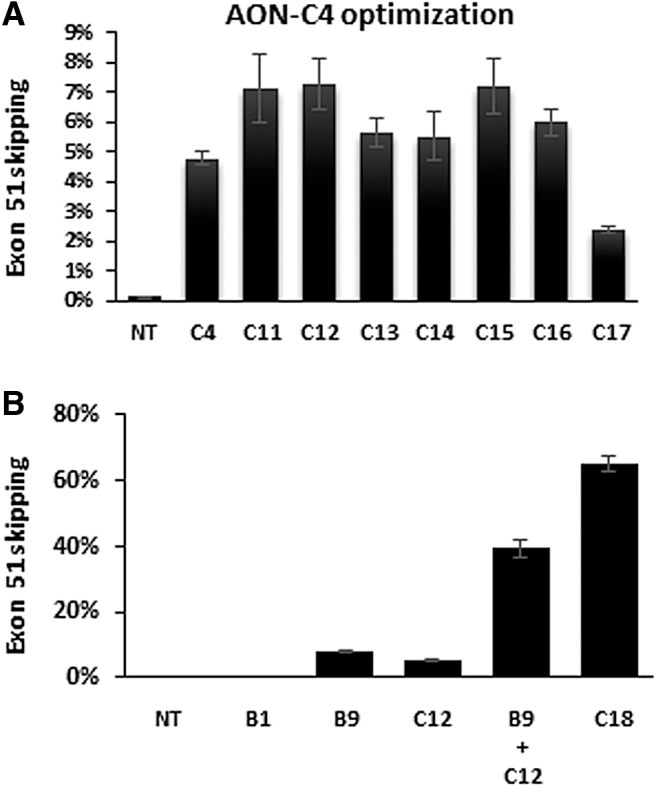
*In vitro* screening of AONs targeting box C through gymnotic uptake in muscle cell cultures from a DMD patient with a deletion of exons 48–50. RT-ddPCR results, skip % of total *DMD* transcripts, mean of *n* = 3 ± SD. **(A)** Identification and optimization of AON-C4 by fine-tuning LNA content and position. **(B)** The synergistic effect of targeting both box B and box C by a mixture or linked AON-B9 and AON-C12 (AON-C18).

Only the AONs that spanned box C induced detectable exon 51 skipping (up to 5% for AON-C4). AON-C4, a 2′-*O*-methyl PS RNA AON with 5-methylcytosines and 5′ and 3′ LNAs, was thus further optimized for LNA content and positions, implementing one or two 5′ terminal guanine LNAs, a 3′ terminal cytosine-LNA and/or one or two additional internal guanine LNAs (Fig. 1B). Comparative testing of these AONs at 800 nM (in a similar experimental setup as in Fig. 2A) resulted in the identification of three AONs (AON-C11, AON-C12, and AON-C15) with the highest exon 51 skipping efficacy (Fig. 4A). Of these AONs with slightly different LNA profiles, AON-C12 was most efficient (7.3%). In subsequent *in vitro* comparative screening assays, AON-C12 was found to be 10-fold more effective than AON-B1.

Following the identification of this second effective target site (box C), we also investigated the effect of double targeting [42], treating cells with two AONs that target different sites within exon 51 to improve skipping efficiencies. A mixture of AON-B9 (box B) and AON-C12 (box C; 800 nM each) was tested in DMD patient (del 48–50) myotube cultures (similar experimental setup as in Fig. 2A). Compared to each AON alone (AON-B9: 8%, AON-C12: 5.2%), a synergistic increase of exon 51 skipping to 39.3% was obtained (Fig. 4B). Linking both AONs with a spacer (AON-C18, Fig. 1B) remarkably improved the efficiency even further to 65.2% exon 51 skipping.

### *In vivo* screening of AONs targeting box C in the hDMDdel52/*mdx* mouse model

Based on these results, we selected AON-C12 and AON-C18 for further screening in the hDMDdel52/*mdx* mouse model. We also included a modified version of AON-C12, containing a 5′-terminal spacer (AON-C19), which is, as such, the 3′ part of AON-C18. In this more extensive study (compared to that in Fig. 3C), we included assessment of AON tissue levels, exon 51 skipping and corresponding dystrophin levels, serum biomarkers, body weight, routine clinical chemistry/hematology, histopathology, and kinematic gait profiling (for AON-C19 only), with comparison to vehicle-treated hDMDdel52/*mdx* and C57BL6/J mice when relevant.

Given the 10-fold higher *in vitro* efficiency of AON-C12 (compared to AON-B1), a lower dose was used in the subsequent *in vivo* study in hDMDdel52/*mdx* mice. Mice received 13 weekly tail vein injections of AON-C12 and AON-C19 at an equimolar dose of 18 mg/kg. AON-C18 was dosed at 19.8 mg/kg (which is equimolar to a mixture of 9 mg/kg AON-C12 and 9 mg/kg AON-B9, so to a total of 18 mg/kg of both AONs combined).

**FIG. 5.**
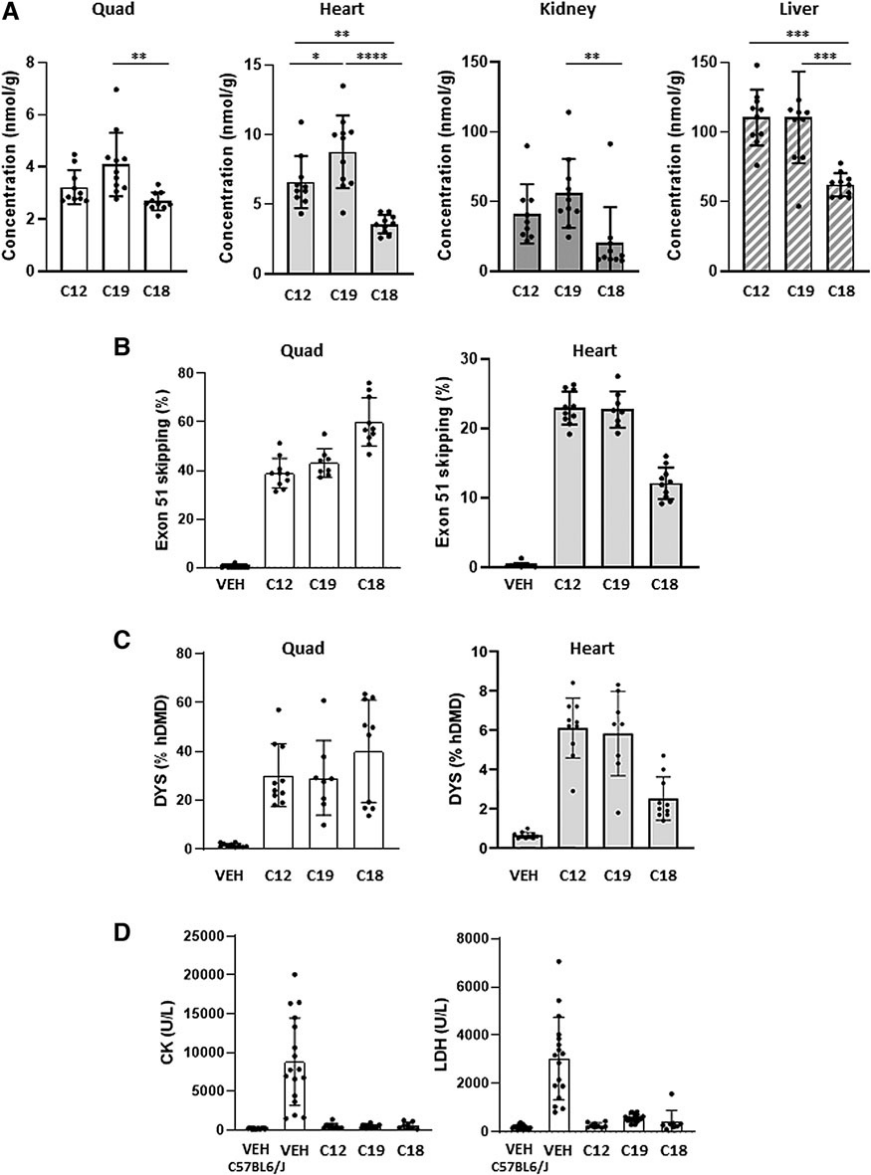

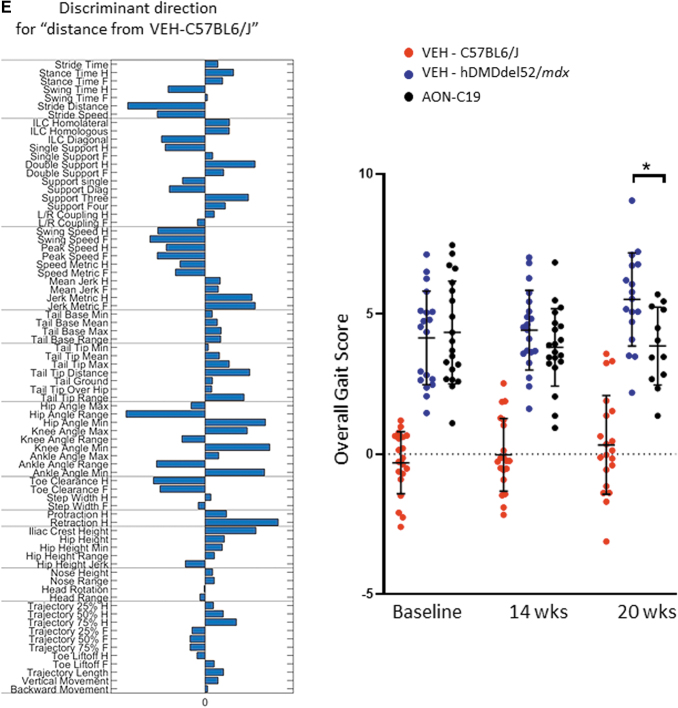
*In vivo* screening of AONs targeting box C in the hDMDdel52/*mdx* mouse model. All values are presented as mean ± SD. *Dots* represent values obtained in individual mice (*n* = 8–10). **(A)** AON concentrations (nmol AON per gram tissue) in quadriceps, heart, kidney, and liver. Significance was assessed using ANOVA and Tukey's Multiple Comparisons test (*P < 0.05, **P < 0.01, ***P < 0.001, ****P < 0.0001). **(B)** Exon 51 skipping levels in quadriceps and heart (RT-ddPCR, skip % of total *DMD* transcripts). **(C)** Dystrophin levels (WES, dystrophin % of healthy hDMD). Note: the *Y*-axis scale of the graphs in **(A–C)** is different for the tissues analyzed to focus on the differences between the AONs. **(D)** Levels of serum biomarkers CK and LDH. **(E)** MotoRater analysis of VEH-treated C57BL6/J and hDMDdel52/*mdx* mice compared to AON-C19-treated hDMDdel52/*mdx* mice (for each cohort *n* = 20), at 14 and 20 weeks of age (after 8 and 13 doses, respectively). Discriminant vector representing the overall kinematic fingerprint of, and the differences between, C57BL6/J and hDMDdel52/*mdx* mice (*left*). Overall Gait Score, based on the established discriminant vector (*right*). Values are presented as mean ± SD. Significance between VEH- and AON-C19-treated hDMDdel52/*mdx* mice was assessed using two-way mixed-effects ANOVA and Tukey's multiple comparisons test (*P < 0.05). ANOVA, analysis of variance; CK, creatine kinase; LDH, lactate dehydrogenase; U/L, units per liter. VEH, vehicle-treated hDMDdel52/*mdx* mice (*n* = 20); VEH-C57BL6/J, VEH-treated C57BL6/J (WT) mice (*n* = 20); VEH, vehicle; WES, Simple Western Analysis; WT, wild-type.

AON tissue levels were determined by ELISA, not only in quadriceps and the heart but also in off-target tissues kidney and liver (Fig. 5A). For AON-C12 and AON-C19, similar concentrations were measured in all four tissues. The tissue levels of AON-C18 (a 38-mer) were, however, significantly lower, which may relate to a reduced, length- and/or PS-dependent, uptake. Noteworthy, for all three AONs, the concentrations in the heart were higher than in quadriceps, which may relate to the different muscle tissue structure and correlated uptake, and/or sequestration in interstitial tissue.

AON-C12 induced 38.9% exon 51 skipping in quadriceps and 22.9% in the heart (Fig. 5B). This is a 65-fold improvement over the levels obtained with AON-B1 at 60 mg/kg in this mouse model (Fig. 3C). Similar efficiencies were obtained with AON-C19, which correlates with the relative tissue concentrations. Noteworthy, despite the relatively higher AON-C12 and AON-C19 concentrations in the heart, the corresponding exon skipping levels were almost twofold lower. This observation may relate to the differential expression and function of dystrophin in heart [43]. Remarkably, despite its lower concentration in quadriceps, AON-C18 induced highest levels up to 61.5% skipping (100-fold better than AON-B1 in Fig. 3C). In the heart, however, AON-C18 was less efficient (12.8%) than AON-C12 and AON-C19, and more in line with the relative tissue concentrations.

The corresponding dystrophin protein levels were quantified by capillary Western immunoassay [13]. Mean dystrophin levels in quadriceps were 30.3% (AON-C12), 29.1% (AON-C19), and 40.1% (AON-C18) of healthy hDMD mouse levels (Fig. 5C). In the heart, respectively, 6.1%, 5.8%, and 2.5% dystrophin restoration was obtained. Exon skipping and dystrophin levels thus correlated well. Immunofluorescence analysis of a limited sample collection (three mice per AON group) confirmed dystrophin expression at the quadriceps muscle fiber membranes (Supplementary Fig. S1).

Next, serum biomarker levels CK and LDH were assessed, which are typically elevated in DMD patients and dystrophic mouse models [9,44,45], whereas CK and LDH levels were indeed highly elevated in vehicle-treated hDMDdel52/*mdx* mice; these were almost reduced to WT levels in mice treated with all three AON candidates (Fig. 5D). The lowering of both serum biomarkers suggests that dystrophin levels of 30% to 40% of WT (hDMD) levels are sufficient to almost fully resolve the leakiness of muscle fibers in hDMDdel52/*mdx* mice.

To further confirm that the obtained levels of dystrophin also improved muscle function, we applied the highly sensitive MotoRater system [9,35,38] to evaluate gait characteristics and fine motor skills in walking mode of AON-C19-treated versus vehicle-treated hDMDdel52/*mdx* mice and C57BL6/J mice (Fig. 5E). In a previous study, a clear and highly significant difference was observed between (untreated) hDMDdel52/*mdx* mice and C57BL6/J controls [35]. hDMDdel52/*mdx* mice were analyzed at baseline (6 weeks) and after 8 and 13 weekly injections of 19.8 mg/kg AON-C19.

A discriminant vector was established based on those gait features, which demonstrated a large effect size (Fig. 5E, left panel). The vector can be seen as an overall kinematic fingerprint characterizing all relevant gait changes that differ between the different groups of WT and hDMDdel52/*mdx* mice, including stride distance, swing speed, hip, knee, and ankle angles and ileac crest height. At week 20, a significant shift toward WTs was observed in the overall gait discriminant score in AON-C19-treated mice relative to vehicle-treatment (*P* < 0.05) (Fig. 5E, right panel).

Finally, at the doses tested in this study, AON-C12, AON-C19, and AON-C18 appeared to be similarly well tolerated, with no effect on survival, body weight, routine clinical chemistry, or hematology. In general, no or only minimal histopathological changes were observed in kidney, liver, spleen, lymph nodes, skeletal muscle, and heart. The histopathological observations were consistent with the well-known class effects of phosphorothioate oligonucleotides [46]. In the kidney, minimal basophilic granules were observed in the cytoplasm of epithelial cells lining the renal tubules in 1 out of 10 mice given AON-C18. In the liver, hepatocellular single-cell necrosis was observed in 6 out of 11 mice given AON-C19 and the severity was generally minimal. In addition, minimal hepatocellular single-cell necrosis was observed in one out of 10 mice given AON-C12 and 2 out of 10 mice given AON-C18, but not in any vehicle-control mice of either gender.

In the heart, myocardial fibrosis/fibroplasia was observed at a slightly higher incidence and/or severity in treated groups than in the control group, especially in males given AON-C18. Any relationship between these histopathological changes and AON treatment was unclear, although it could not be excluded. All other histopathological findings in the heart were observed at similar incidence/severity in control and treated groups and were therefore considered unlikely to be related to AON-treatment.

In skeletal muscle, fiber necrosis, muscular atrophy, basophilia/regeneration, inflammation, mineralization, and fatty changes were observed as part of the expected morphological appearance of the skeletal muscle in this DMD mouse model (Supplementary Table S1). After treatment with each of the AONs (AON-C12, AON-C18, or AON-C19), there was a reduction of incidence and/or severity of muscle fiber atrophy, necrosis, and inflammation compared to vehicle-treated mice, which may relate to the restored dystrophin expression.

## Discussion

Drisapersen, with a fully modified 2′-*O*-methyl PS RNA chemistry, was the first AON taken in clinical development for DMD in 2006 [47]. Its target sequence located at position (+68 + 87) in dystrophin exon 51 (Box B in Fig. 1) was hypothesized to be involved in the correct splicing of the exon through interaction with splicing factors like the SR proteins [48,49]. Binding of drisapersen to its target in exon 51 indeed induced exon 51 skipping from the dystrophin pre-mRNA, most likely by sterically hindering binding of splicing factors. However, with the nonquantitative methods used in those days [nested reverse transcriptase polymerase chain reaction (RT-PCR) and western blot analysis], its efficiency to do so may have been overestimated.

Furthermore, an extensive and costly AON candidate selection screening to identify more potent molecules was not feasible in drisapersen's early phase academic setting. As pioneering compound in the field, it was also unknown what levels of exon 51 skipping would be required for restoring dystrophin expression at sufficient levels to achieve a clinically relevant effect. Ten years and multiple clinical studies later, it became evident that drisapersen, as well as its analogs in the field targeting the same box B site in exon 51 (eteplirsen and suvodirsen), was not as efficient as hoped for [15,18].

Based on their relative simplicity, safety (transient effect on RNA level), and manufacturing costs compared to the alternative gene therapy approaches, oligonucleotides remain attractive drug candidates. Supported by a large amount of data available from all the preclinical and clinical studies, lessons learned are available for implementation to improve the design, chemistry, pharmacokinetic profile, efficiency, and safety of next generation AONs.

Starting point is design, followed by more extensive AON candidate screening, both *in vitro* and *in vivo* clinically relevant DMD models, and using more accurate and quantitative methods (such as ddPCR [9,37] and WES ProteinSimple analysis [9,13]), to select the most efficient ones for further nonclinical development. Our aim was to identify (2′OMePS) RNA AON candidates with at least 10-fold better *in vivo* efficiency than drisapersen (AON-B1), resulting in a more favorable therapeutic index. Two lines of investigation were followed: varying length and chemical composition of AONs compared to AON-B1 and identification of an alternative more potent site involved in exon 51 splicing.

Over a hundred 2′OMePS RNA AONs were screened in differentiated DMD (Δ48–50) muscle cell cultures, targeting either the AON-B1 (box B) or alternative target site (box C). Most efficient AONs (over 20 AONs in total) were selected for further comparative analysis in hDMD and hDMDdel52/*mdx* mice. With regard to design and chemical composition, the natural 5-methylcytosine substitution was standardly implemented in each AON, as in multiple FDA-approved AONs [29]. LNAs were implemented to enhance resistance to exonucleases and increase AON-target duplex stability. The positive effects of LNAs on AON efficiency are well known in the field [50–53]. We, however, limited the number of LNAs per AON (preferably one or two, not more than 6) to avoid any reduced specificity [54] or safety (as observed for RNaseH-activating gapmers [52,55]).

Despite the LNAs, AONs shorter than 19 nucleotides were not sufficiently efficient, that is, not (much) more or even less efficient than AON-B1. We also included longer AONs (21 or 22 nucleotides) with LNAs, with the idea that more efficient AONs would allow lower dosing, which also contributes to a favorable safety profile. AON-B2, however, a 22-mer with one 5′-terminal LNA, was selected for analysis in the first hDMDdel52/*mdx* study, but needed higher dosing to meet the threshold of 10-fold higher efficiency than AON-B1. In retrospect, inclusion of AON-B4, a 22-mer with 4 LNAs, in that study would likely have been more informative.

The implementation and fine-tuning of LNAs at specific positions had strong impact. Especially, when positioned at the 5′ and/or 3′ termini, one or two LNA-nucleosides could already improve exon skipping efficiencies over 10-fold, in part, due to increased AON-target RNA duplex stability, but likely also due to reduced sensitivity to exonucleases. This hypothesis was indeed supported by *ex vivo* stability studies using human recombinant exonucleases (data not shown). While acknowledging the limitations of the designed set of AONs (wherein not every position for an LNA nucleoside was analyzed), the positive effect of internal guanine-LNAs was evident, whereas the implementation of, for instance, internal adenine-LNAs seemed of limited benefit.

The most efficient AON in the studied series targeting box B was AON-B9, a 20-mer with a 5′- and 3′-terminal thymine-LNA and an internal guanine-LNA, which was 15-fold more efficient than AON-B1 at the same dose of 60 mg/kg (Fig. 3C). In the *in vivo* studies in hDMD and hDMDdel52/*mdx* mice, the presence of LNAs did not lead to any safety concern, but more in-depth safety studies need to be performed to assess this in more detail.

It is remarkable that more than 10 years and multiple clinical studies later, the main clinical exon 51 skipping AONs reported to date have targeted the same upstream box B site in exon 51. Since then, it has become evident that not only first in class drisapersen but also its later analogs eteplirsen and suvodirsen with (almost) identical sequences were not as efficient as hoped for [15,18].

In this study, we performed a thorough re-screening of the entire exon 51 sequence for potential splicing regulatory sites and identified a novel more potent locus in exon 51 (box C), allowing the use of shorter and more effective AONs like AON-C12 (18-mer). AON-C12 (and its AON-C19 analog), an 18-mer with two 5′-terminal and one internal guanine-LNAs and a 3′-terminal cytosine-LNA, induced 65-fold higher exon skipping levels in quadriceps at 18 mg/kg (Fig. 5B) compared to a threefold higher dose of AON-B1 (60 mg/kg) (Fig. 3C).

Simultaneous targeting of both box B and C by treating DMD patient cell cultures with a mixture or linked combination of AON-B9 and AON-C12 (AON-C18) was clearly synergistic, with exon 51 skipping levels up to 65% (8-fold higher than each AON alone). Despite the fact that AON-C18 is a more complex and longer (38-mer) molecule with potential safety liabilities related to the relatively high number of PS linkages and LNAs, such increased efficacy justified inclusion in the second hDMDdel52/*mdx* screening study, although at a relatively lower dose.

Indeed, at a dose equimolar to 9 mg/kg AON-C12, AON-C18 induced up to 61.5% exon 51 skipping (100-fold higher than with AON-B1 at 60 mg/kg), and seemed well tolerated by the mice (based on body weight and survival). Although the difference in exon 51 skipping levels between AON-C12 (at 18 mg/kg) and AON-C18 (at 9 mg/kg equimolar) was large (38.9% and 61.5%, respectively), the relative increase in restored dystrophin levels with AON-C18 was smaller (30.3% and 40% of healthy control, respectively). In that respect, the shorter and less complex AON-C12 (or its AON-C19 equivalent) may still be more favorable for further (pre)clinical development. Alternatively, further reduction of length and LNA content of AON-C18 may lead to at least equally efficient AONs that can be dosed at higher levels without safety issues.

Interestingly, in a recent other AON screening study focusing on phosphorodiamidate morpholino oligomers [56], this box C region was not identified as an effective target site for exon 51 skipping. The most efficient morpholino oligomers transfected into immortalized muscle cell cultures in fact covered the 5′ site of exon 51 (upstream of box B, Fig. 1A), which was not considered an interesting target in our studies. This discrepancy may relate to the difference in backbone chemistries (2′OMePS RNA vs. phosphorodiamidate morpholino oligomers), transfection methods (gymnotic uptake vs. Endo-porter transfection reagent), and/ or RT-PCR technologies (RT-PCR vs. RT-ddPCR).

Furthermore, the conclusions drawn from the morpholino oligomer screening study [56] were primarily based on *in vitro* data, with only one compound further tested in hDMD mice by intramuscular injections in the tibialis anterior muscle, and assuming predictability from *in vitro* DMD patient cell cultures to *in vivo* humanized DMD mouse models. For most 2′OMePS RNA AONs in our studies that was indeed the case; for instance, AON-B9 and AON-B2 were the most efficient compounds, both in the DMD patient cell cultures and the hDMD and hDMDdel52/*mdx* mouse models. However, the remarkable level of improvement obtained with AON-C12 in hDMDdel52/*mdx* mice was not foreseen based on the more moderate *in vitro* results (Fig. 4). Obviously, other factors like biodistribution and muscle fiber uptake can play an additional differentiating role as well.

Nevertheless, the *in vitro* prescreening is a useful and relatively quick tool facilitating a high-level selection of the most promising candidates from a large series of AONs, but that selection should not be too stringent and thus exclude AONs that are potentially more effective *in vivo*. Despite animal ethics, costs, and timelines, the *in vivo* screening should thus not be skipped and include multiple lead AON candidates tested in at least 5, but preferably 10–15, mice per cohort to take animal variation into account as well. With such extensive screening studies, it is neither practical nor logistically feasible to analyze multiple muscle groups per mouse and implement all currently available dystrophin-related (histological or functional) outcome parameters.

Previous *mdx* mouse studies on M23D indicated that for 2′OMePS AONs, the corrective effect in quadriceps is representative for that in other skeletal muscle groups, including diaphragm [9,57]. With the primary aim in mind (to identify, compare, and select new and more favorable AON candidates for *DMD* exon 51 skipping), we therefore also focused on relatively high-throughput, quantitative assays to assess AON tissue concentrations (ELISA), exon skipping (RT-ddPCR), and dystrophin levels (capillary Western immunoassay), in quadriceps and the heart (considering its distinct muscle tissue architecture) only.

The studies described herein were performed over a period of more than 3 years and initially the hDMDdel52/*mdx* mouse model was not available. Therefore, the first *in vivo* comparative AON candidate studies were done in the healthy, nondystrophic hDMD mouse model. Although limited by a lower muscle tissue uptake and exon skipping levels as single readout, we were already able to select the most efficient AONs like AON-B9, which also turned out to be most efficient for box B in the follow-up studies in hDMDdel52/*mdx* mice. This latter humanized model clearly is more clinically relevant and allows final selection of human AON lead candidates before further nonclinical development in nonhuman primates. It was generated using transcription activator like effector nuclease (TALEN) technology, and recently further characterized on genetic and functional level [35].

Although the genomic composition of the *hDMD* and *hDMDdel52* transgenes in this model appeared more complex than initially expected, that does not change its suitability for comparative screening of AONs inducing exon 51 skipping. Functional characterization revealed a clear difference in overall gait parameter profile between hDMDdel52/*mdx* mice and C57BL6/J controls [35] (Fig. 5E).

Treatment with AON-C19 for only 13 weeks induced dystrophin levels up to 29% dystrophin of healthy (hDMD) control levels, which already resulted in a significant shift for a series of gait parameters toward C57BL6/J. In fact, the obtained levels of dystrophin are within the same range (from 10% to 90%, with a mean of 33% of the healthy muscle) as previously reported for muscle biopsies from patients diagnosed with the typically milder BMD [13]. These BMD patients express truncated dystrophin proteins resulting from in-frame mutations in the *DMD* gene, and resembling those resulting from AON-induced exon skipping.

In a previous *mdx* study [9], we compared the efficacy of subcutaneous (SC) versus IV dosing routes. With a similar set of state-of-the-art quantitative technologies, including ddPCR, capillary Western immunoassay, and automated kinematic analysis, it was concluded that IV dosing of the AON had a more pronounced beneficial effect, both at the molecular and functional level, compared to SC dosing. Based on these results, we decided to use IV dosing in the hDMD and hDMDdel52/*mdx* studies described herein as well. Furthermore, based on the adverse injection site reactions in the drisapersen clinical studies following SC administration, we foresee IV administration in future clinical studies on next generation AONs. This is supported by a recent overview of absorption, distribution, metabolism, and excretion of 2′-*O*-methyl PS AONs, including drisapersen, compiled from publicly available data and previously unpublished data on drisapersen and related exon skipping candidates in preclinical species and DMD patients [57].

In conclusion, we here report on a novel and more potent target site in *DMD* exon 51 for induction of exon skipping. By targeting this novel site with AONs containing chemical modifications like 5-methylcytosine and LNAs at specific positions, we succeeded in designing next generation AONs that induced much higher, and more clinically relevant, levels of exon skipping (up to 60%) and restored dystrophin expression (up to 40%) compared to drisapersen. Such markedly increased efficiencies may allow for lower clinical dosing, and thus improved safety. AON-C12, AON-C19, and AON-C18 are therefore interesting lead molecules for further nonclinical development (including more extensive comparative safety screening studies in rodents and nonhuman primates), and their improved therapeutic index may pave the way toward more effective future AON therapies for DMD.

## Supplementary Material

Supplemental data

Supplemental data
