# Supplementary material for: Next Generation Exon 51 Skipping Antisense Oligonucleotides for Duchenne Muscular Dystrophy
Source: Nucleic Acid Ther. 2023 Jun 2;33(3):193–208. doi: 10.1089/nat.2022.0063 (PMC10277991; doi:10.1089/nat.2022.0063)
Supplement: Supplemental data [file Suppl_FigureS1.pdf]

Supplementary Figure 1. Immunofluorescence analysis of dystrophin expression

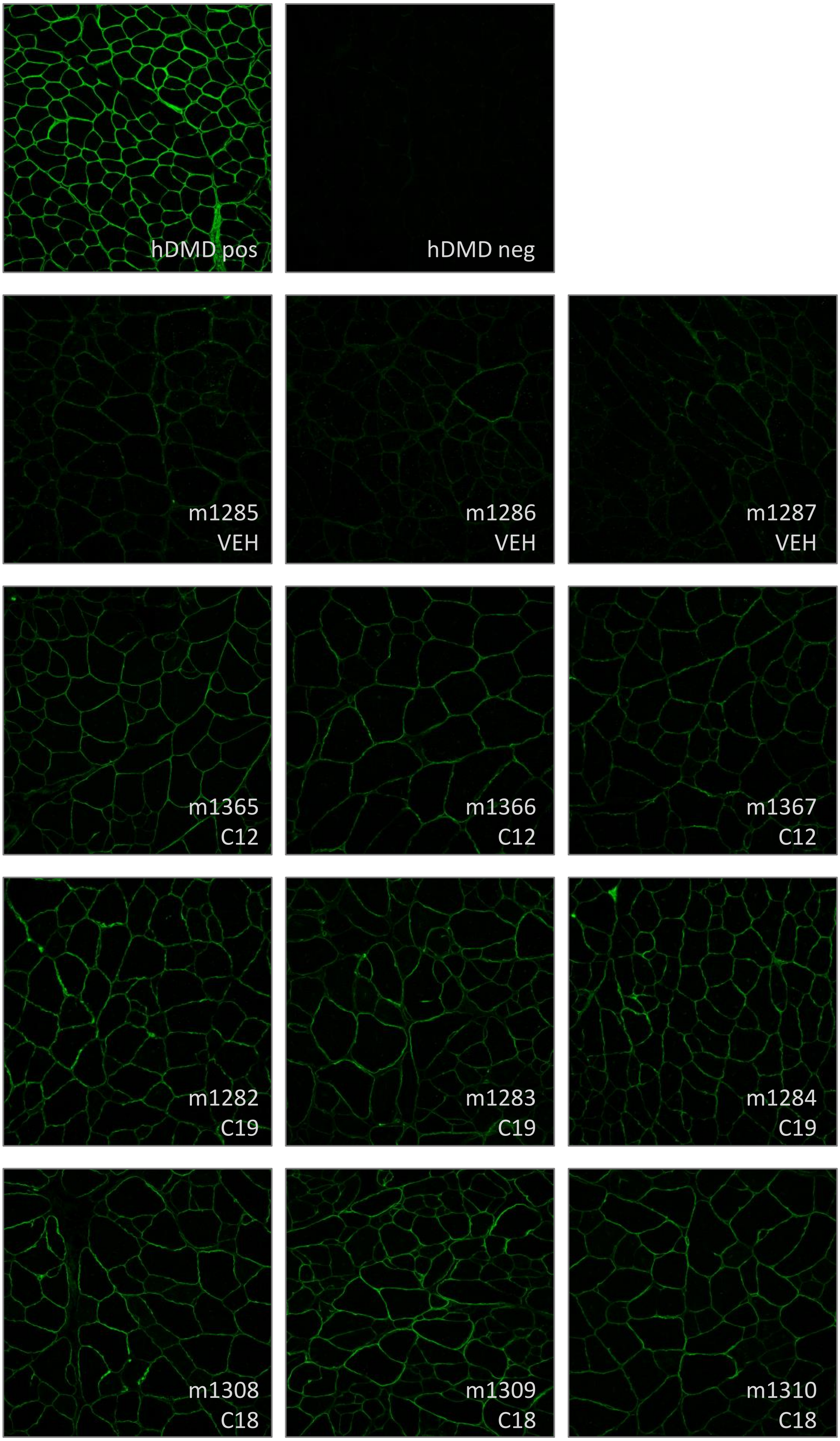

Supplementary Figure 1. Immunofluorescence analysis of dystrophin expression at the muscle fibre membranes. Cryosections (8 µm) from quadriceps muscles were mounted on Superfrost Ultra Plus microscopy slides (Fisher Scientific), and incubated for 2 hr with primary antibody rabbit polyclonal anti-dystrophin (Ab15277, Abcam, dilution 1/200). Slides were rinsed and washed twice for 5 minutes in PBS and subsequently incubated for 1 hr with secondary antibody goat-anti-rabbit AlexaFluor488 (ThermoFisher) in a dilution of 1/250. Imaging of slides was performed on the same day on a Zeiss LSM 710 confocal microscope using a 25x objective and laser intensity 6%. Shown are images from 3 different mice per VEH (vehicle) or AON group. Upper panel: control sections from a non-dystrophic hDMD mouse (pos = with dystrophin primary antibody Ab15277, neg = with rabbit IgG isotype primary antibody Ab27478).
