# Supplementary material for: Next Generation Exon 51 Skipping Antisense Oligonucleotides for Duchenne Muscular Dystrophy
Source: Nucleic Acid Ther. 2023 Jun 2;33(3):193–208. doi: 10.1089/nat.2022.0063 (PMC10277991; doi:10.1089/nat.2022.0063)
Supplement: Supplemental data [file Suppl_TableS1.docx]

**Supplementary Table 1. Incidence of relevant histological findings in skeletal muscle**

| **AON** | **Vehicle** | **C12** | **C19** | **C18** |
| --- | --- | --- | --- | --- |
| **No. of Male/Females** | **6/4** | **6/4** | **7/4** | **5/5** |
| **Muscle fibre atrophy** |  |  |  |  |
| Minimal | - | 2/1 | 1/2 | - |
| Mild | -/2 | 3/3 | 4/2 | -/5 |
| Moderate | 4/1 | - | 2/- | 4/- |
| Marked | 2/1 | - | - | 1/- |
| **Muscle fibre mineralization** |  |  |  |  |
| Minimal | 5/3 | 4/1 | 2/2 | 3/4 |
| Mild | 1/1 | 1/2 | 5/2 | 1/- |
| **Fatty change** |  |  |  |  |
| Minimal | -/1 | -/2 | 1/3 | 1/3 |
| **Muscle fibre necrosis** |  |  |  |  |
| Minimal | 2/2 | 2/- | 4/1 | 1/- |
| Mild | 3/- | - | - | - |
| Moderate | -/1 | - | - | - |
| Marked | -/1 | - | - | - |
| **Basophilia/regeneration** |  |  |  |  |
| Minimal | 3/3 | -/1 | 1/- | - |
| Moderate | -/1 | - | - | - |
| **Inflammation** |  |  |  |  |
| Minimal | 6/2 | - | -/1 | - |
| Moderate | -/1 | - | - | - |

-: finding not observed in mice in the group.
